# *Inter*‐ and *Intra*‐Molecular Charge Redistributions in H‐Bonded Cyanuric Acid*Melamine (CA*M) Networks: Insight From Core Level Spectroscopy and Natural Bond Orbital Analysis

**DOI:** 10.1002/chem.202403782

**Published:** 2025-01-07

**Authors:** Daniele Toffoli, Roberto Costantini, Elisa Bernes, Lorys Di Nardi, Gabriele Balducci, Elisa Viola, Giovanna Fronzoni, Albano Cossaro, Valeria Lanzilotto

**Affiliations:** ^1^ Dipartimento di Scienze Chimiche e Farmaceutiche Università degli Studi di Trieste 34127 Trieste Italy; ^2^ IOM-CNR Istituto Officina dei Materiali-CNR S.S.14, Km 163.5 34149 Trieste Italy; ^3^ Dipartimento di Fisica, Università degli Studi di Trieste 34127 Trieste Italy; ^4^ Dipartimento di Chimica, Sapienza Università di Roma 00185 Italy

**Keywords:** charge transfer, hydrogen bonds, density functional calculations, nitrogen heterocycles, photoelectron spectroscopy

## Abstract

In this work, we elucidate the electronic charge redistributions that occur within the cyanuric acid (CA) and melamine (M) molecules upon formation of the triple H‐bond between the imide group of CA and the diaminopyridine group of M. To achieve this, we investigated 2D H‐bonded assemblies of M, CA and CA*M grown on the Au(111) surface, using X‐ray photoemission (XPS) and near edge X‐ray absorption fine structure (NEXAFS) spectroscopies. Compared to the homomolecular networks, the spectra of the mixed sample reveal core level shifts in opposite directions for CA and M, indicating a nearly complementary charge accumulation on the CA molecule and a charge depletion on the M molecule. These findings were further confirmed by theoretical simulation of the ionization potentials (IPs), which were computed using unsupported models of the H‐bonded networks. A natural bond orbital (NBO) analysis performed on the three systems helped to rationalize the net charge transfer form M to CA. Finally, we observed that intramolecular interactions (electron delocalization effects) contribute progressively to the charge redistributions inside the two molecules when going from the homomolecular to the heteromolecular networks.

## Graphical abstract

The heteromolecular H‐bonding interaction between Cyanuric Acid (CA) and Melamine (M) involves two types of H‐bonds, where the two molecules alternatively act as H‐donors and H‐acceptors. However, the strengths of these interaction are unequal resulting in a net charge transfer from M to CA. Additionally, the intermolecular charge transfer is accompanied by enhanced electron delocalization within both molecules.

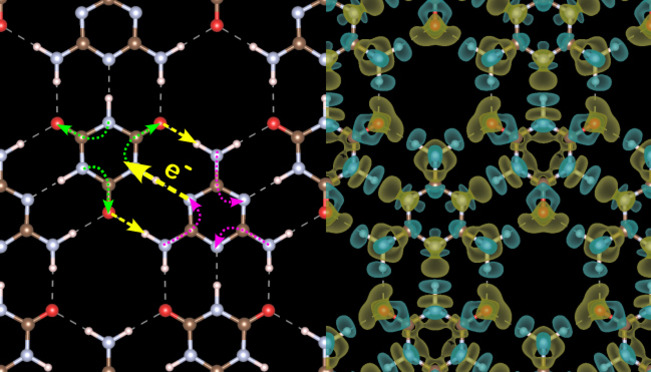

## Introduction

**Figure 1:**
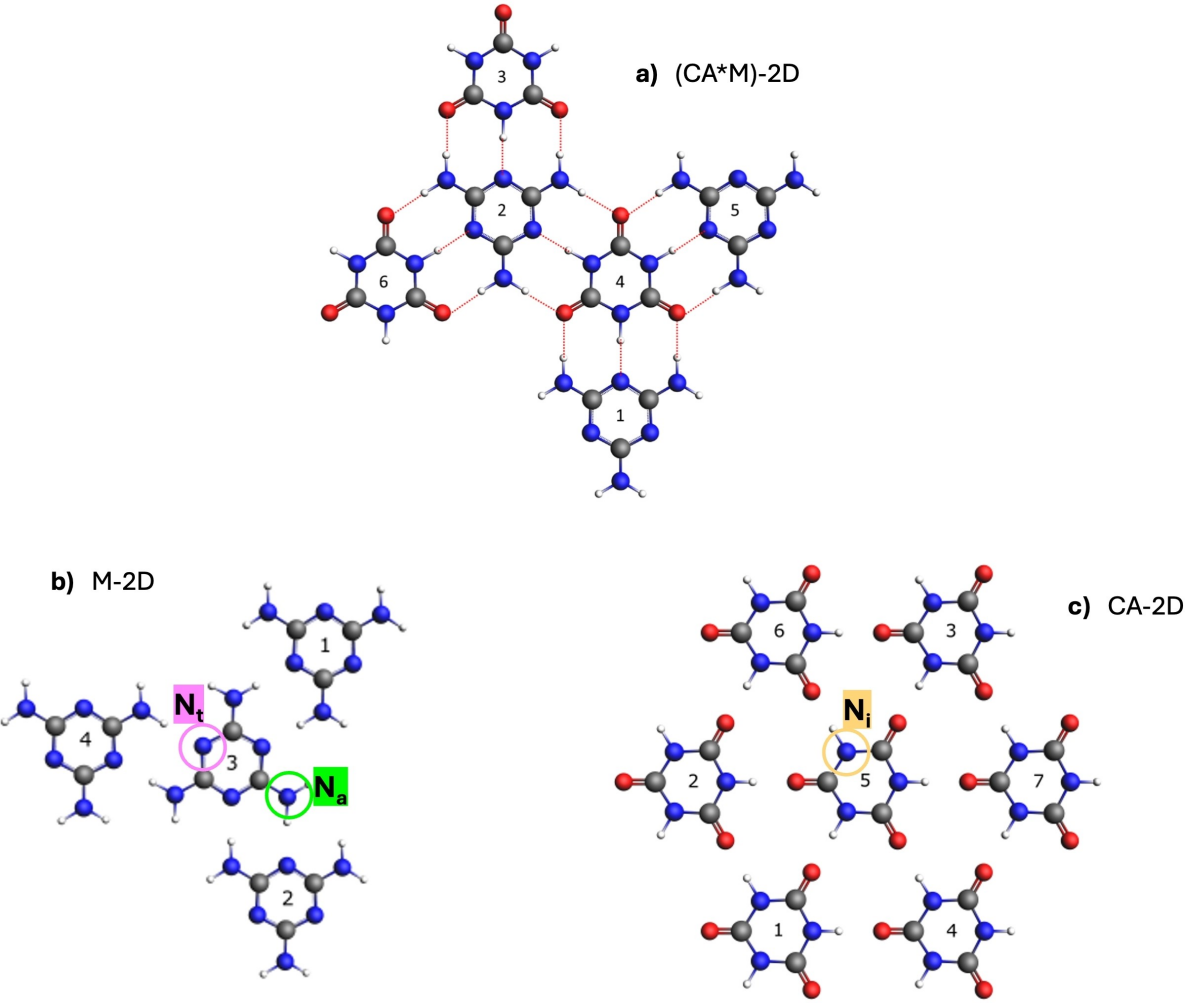
Chemical structure of the H‐bonded models used to simulate the bimolecular and the homomolecular H‐bonded networks grown on the Au(111) surface. a) (CA*M)‐2D model; b) M‐2D model and c) CA‐2D model. C atoms are in grey, N atoms in blue, O atoms in red and H atoms in white. N_t_ and N_a_ indicate the triazine‐N and amino‐N atoms of M, N_i_ indicates the imide‐N atom of CA. A numbering of the molecular units within each H‐bonded system, used in Tables S1‐S6 of the SI, is also included.

Since the pioneering work of *G. M. Whitesides*,[^1^, ^2^, ^3^] who provided excellent examples of *rosette*‐ and *tape*‐ structures based on the cyanuric acid‐melamine (CA*M) triple hydrogen bond (see Figure 1a), the non‐covalent CA*M interaction has been continuously exploited in areas such as co‐crystals engineering,^[4]^ sensing and biosensing.[^5^, ^6^, ^7^] Most recently, novel layered materials with tunable optoelectronic properties have been developed using pre‐formed 2D CA*M sheets as templates to drive the adsorption of various functional organic molecules (i. e. trimesic acid, terephthalic acid, PTCDA or C_60_).[^8^, ^9^] Another attractive exploitation of the bidimensional CA*M network involves its integration with 2D semiconductors^[10]^ or inorganic nanoparticles,^[11]^ providing an enhancement of their electrocatalytic activity for hydrogen production.

In contrast to the prolific literature on CA*M applications, there are far fewer fundamental studies about the intrinsic electronic properties of the system.[^12^, ^13^, ^14^] Knowledge of such properties may be relevant to fully understand the interfacial phenomena the system may be involved in, such as its role as template in the epitaxial growth of aromatic carboxylic acids,^[8]^ or its ability to boost the electrocatalytic H_2_ evolution activity of metal dichalcogenides.^[10]^ More specifically, there is a lack of a comprehensive description of the electronic charge redistribution within the CA and M molecules following the H‐bonds formation. In this context, our group has recently paid attention on the local electronic state of the N‐functionalities of both CA and M, examining the changes that occur when going from homomolecular networks to the bimolecular one.^[12]^ The study, based on near edge X‐ray absorption fine structure spectroscopy (NEXAFS) measurements and theoretical simulations, revealed an accumulation of electron density on the CA proton donor site (**N_i_**−H) and a nearly complementary depletion at the M proton acceptor site (**N_t_**), with no significant charge density variations around the **N_a_**, the proton donor of M. Similar findings were reported by *Damodaran et al*.,^[13]^ who performed a trinuclear NMR (^2^H, ^13^C, ^15^N) characterization on crystalline samples of CA, M and CA*M. Comparison of the ^15^N NMR spectra, indeed, reveals chemical shifts in opposite directions of approximately −10 ppm for N_t_ and +5 ppm for N_i_. On the other hand, no significant chemical shifts were detected for N_a_. Interestingly, ^13^C NMR spectra also feature complementary chemical shifts of *ca*. −2.5 ppm for the M carbon atoms and +2.5 ppm for those of the CA molecule.

These findings suggest that the two types of hydrogen bonds present in the system, N_i_−H⋅⋅N_t_ and N_a_−H⋅⋅O, are not equivalent, resulting in non‐trivial charge redistributions within the two molecules.

To thoroughly investigate the electronic properties of the CA*M system, we expanded our previous study based on the N K‐edge^[12]^ to the C 1s and O 1s levels by using both X‐ray photoemission (XPS) and NEXAFS spectroscopies. The experimental XPS and NEXAFS spectra were acquired on monomolecular and bimolecular samples grown on the Au(111) surface, and compared with theoretical simulations based on the unsupported H‐bonded models depicted in Figure 1. A natural bond orbital (NBO) analysis was applied to both systems to rationalize the observed trends in measured binding energies (BEs) and to provide detailed insights into the evolution of inter‐ and intra‐molecular charge redistributions upon the formation of the CA*M supramolecular system from its monomolecular counterparts.

## Experimental Details

Measurements were performed at the ANCHOR‐SUNDYN endstation^[15]^ installed at the ALOISA beamline of the Elettra Synchrotron facility (Trieste, Italy). CA (purity 98 %) and M (purity 99 %) molecules were purchased from Mercks and sublimed on the Au(111) substrate from Knudsen cells at temperatures of 423 K and 393 K, respectively. The Au(111) sample was cleaned by Ar^+^ bombardment and annealing (to 770 K) cycles. All samples/films shown herein were prepared by keeping the substrate at room temperature. Growth of the homomolecular samples was characterized by the concurrent adsorption of first and ad‐layer molecules (see models in Figure 3a). According to the work of W. Xu et al.,^[16]^ we chose to grow the bimolecular film by sequential deposition of the CA molecule on top of a pre‐formed H‐bonded M network. This led to the formation of mixed domains but also to the segregation of the individual ones, both in the first and upper layers (Figure 3a). For details about the samples preparation refer to the Supporting Information (SI) File.

XPS and NEXAFS spectra were acquired with a SPECS Phoibos 150 electron analyzer. The O 1s XPS spectra were acquired with photon energy of 650 eV (overall resolution 0.2 eV), while the C 1s XPS spectra were measured with photon energy of 515 eV (overall resolution 0.15 eV). The binding energy (BE) scale was calibrated by aligning the Au 4f_7/2_ photoemission peak to the BE of 84.0 eV. All spectra shown in the main text were measured with emission angle (θ) of 35° and normal incidence. The CA and M XPS spectra were fitted by using Voigt components having common broadening. While the Lorentzian FWHMs were fixed to literature values (0.17 eV for the O 1s, 0.10 eV for the C 1s)^[17]^ the Gaussian FWHMs were left as fit parameters that were found to be 0.81 eV for the O 1s, 0.72 eV for the C 1s of the CA molecule and 0.52 eV for the C 1s of the M molecule. Deconvolution of the spectra of the mixed sample was performed introducing Voigt components with both fixed Lorentzian (literature values) and Gaussian FWHMs. The latter were chosen to be equal to the values found for fitting the spectra of the individual molecules.

O and C K‐edge NEXAFS spectra were measured in Auger yield mode, with photon energy resolution of 0.2 eV and 0.1 eV, respectively. The spectra were collected with the electric field polarization of the light parallel (s‐pol) and almost perpendicular (p‐pol) to the surface plane. The photon energy was calibrated by using the Au 4f_7/2_ line measured with the last photon energy of each NEXAFS spectrum. The p‐pol and s‐pol were normalized to have both spectra with the same pre‐edge and post‐edge intensity.

## Theoretical Details

The free‐standing 2D monolayer of M, CA and CA*M were optimized at the DFT level within the plane‐wave pseudopotential method as implemented in the QUANTUM ESPRESSO (QE) code suite.^[18]^ Details of geometry optimizations of the periodic 2D systems are reported in our previous work.[^12^, ^19^] Finite H‐bonded M, CA and CA*M clusters (M‐2D, CA‐2D and (CA*M)‐2D, see Figure 1) were cut from the periodic structures and used in the subsequent calculations of the XPS and NEXAFS spectra.

The vertical ionization potentials (IPs) were computed at DFT level within the ΔSCF Kohn–Sham (ΔKS) scheme allowing a full relaxation of the ionized core hole. IP^ΔKS^ is given as the difference between the energy of the 1 s^−1^ ionic state, calculated through a Kohn–Sham (KS) spin‐polarized unrestricted scheme, and that of the *N*‐electron configuration.

The NEXAFS spectra at the C and O K‐edges of M and CA*M were calculated within the Density Functional (DFT) transition potential (TP) scheme (DFT‐TP)[^20^, ^21^] which includes the main relaxation effects following the core‐hole formation. At DFT‐TP level, the core‐excitation energies correspond to the energy difference of the final virtual and the core initial TP‐MOs involved in the transition. The DFT‐TP core‐excitation energies are usually too large compared to the experimental ones; to get more accurate values for the excitation energies it is usual to shift the excitation energies by a value corresponding to the difference between the energy of the initial core‐excited TP‐MOs and the IP^ΔKS^ value. This scaling procedure has been applied in this work as well. The hybrid B3LYP *xc* functional[^22^, ^23^, ^24^] was used in the DFT‐TP calculations.

The transition intensities are expressed as oscillator strength *f*_*i→ f*_ which are expressed by the following equation for a fixed in space system:

(1) $$f_{i\to f}=2n_{i}{\Delta E_{i\to f}\left|\langle \phi_{f}^{TP}|\epsilon \;\cdot \mu |\phi_{i}^{TP}\rangle \right|}^{2}$$

where, in Eq. (1), the dipole moment integral involves the initial and final TP MOs, *n*_i_ is the occupation number of the core orbital (equal to two) in the ground state, $\Delta E_{i\to f}$ is the transition energy and **ϵ** is the polarization vector of the incident radiation.

The calculations relative to the finite systems were done by using the Amsterdam Density Functional (ADF) suite of programs.[^25^, ^26^, ^27^] An even‐tempered quadruple‐ζ with three polarization and three diffuse functions Slater‐type orbitals (STO) basis set (ET‐QZ3P‐3DIFFUSE set in the ADF database) was employed for the core‐excited/ionized C and O atoms, to properly describe the diffuse Rydberg‐like states, whereas a frozen‐core triple ζ polarized basis set of STOs (TZP.1 s in the ADF database) was adopted for the nonexcited C, N, and O atoms and a DZP basis set for the H atoms. In case of symmetry‐equivalent atomic centres, a Frozen Core (FC) 1 s basis set was employed to ensure the localization of the core hole.

The natural bond orbital (NBO) analysis[^28^, ^29^, ^30^] uses the donor‐acceptor model of H bonding.[^31^, ^32^, ^33^] In this model the H‐bond interaction B⋅⋅H−A can be described as an electronic charge transfer resulting from the superposition of a high‐energy lone‐pair NBO of the donor (*n*_B_) with an unoccupied low‐lying antibonding NBO of the acceptor (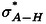 ). The degree of charge transfer/delocalization can be quantified in terms of the perturbative energy lowering 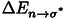 resulting from the orbital overlap, and obtained from standard second order perturbation theory:
(2)

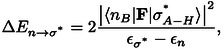




where in Eq. (2), 
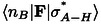
 is the KS matrix element between the donor ($n_{B}$ ) and acceptor (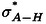 ) NBOs, while the zeroth‐order orbital energies, 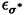 and $\epsilon_{n}$ , of the NBOs enter in the denominator. Alternatively, the charge delocalization due to H‐bond interaction can be expressed in the language of resonance theory as a small weight of the resonance structure II in the resonance hybrid: B: H−A (I)↔B−H^+^: A^−^ (II). The NBO analysis has been conducted with the NBO6 program included with the AMS distribution.

## Results and Discussion

### NEXAFS

**Figure 2:**
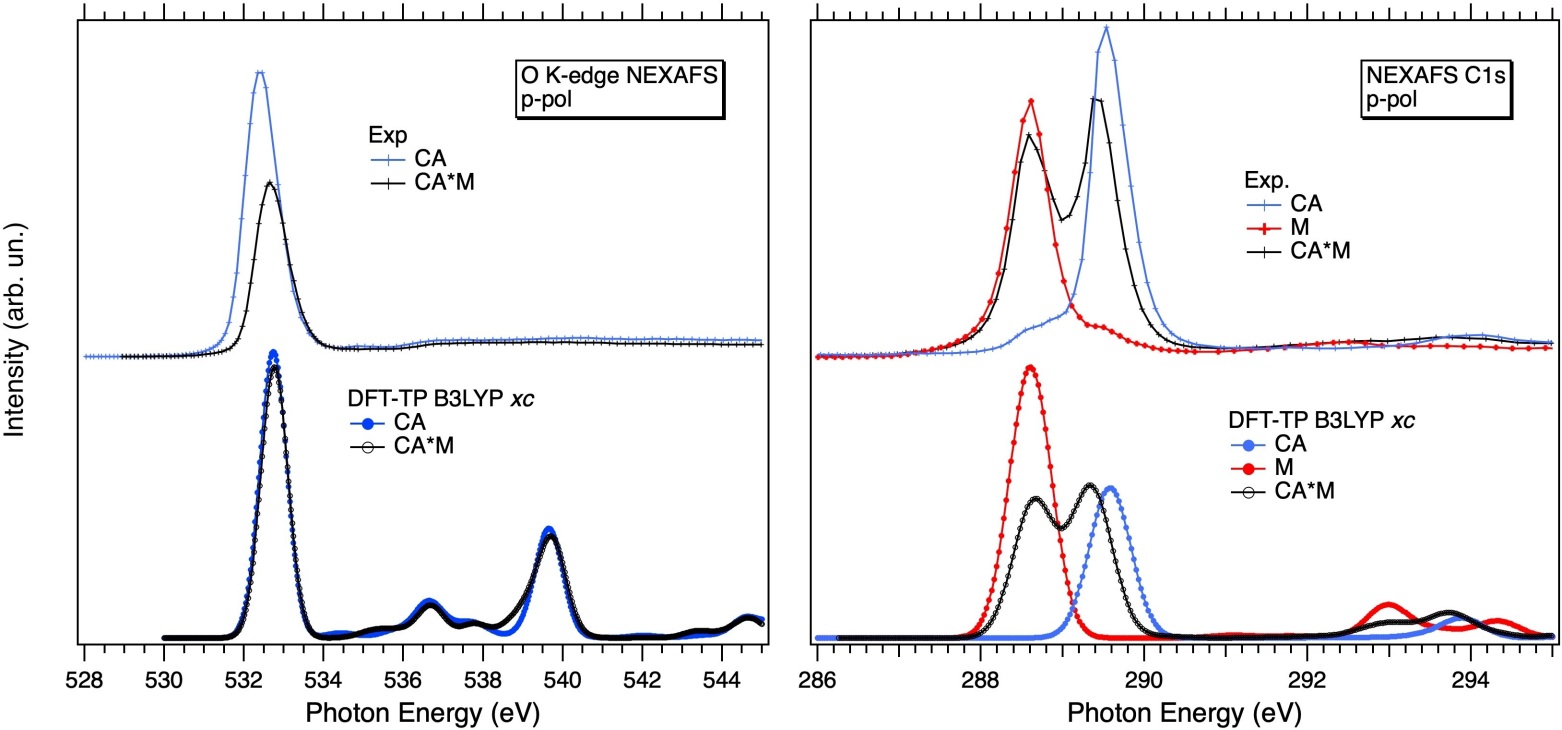
O and C K‐edge spectra (p‐pol) for the mixed sample (CA*M) and the reference samples (CA, M), along with the theoretical counterparts.

In our previous work,^[12]^ we exploited N K‐edge NEXAFS spectra of CA*M and individual CA/M H‐bonded networks grown on the Au(111) surface to extract the fingerprint of the heteromolecular N_i_−H⋅⋅N_t_ H‐bonding interaction between CA and M molecules. Although the CA*M sample was characterized by the co‐presence of heteromolecular and homomolecular domains (both in the first and upper layers), we evidenced that the CA*M interaction has the effect to red‐shift the N_i_ 1 s→LUMO_CA_ and blue‐shift the N_t_ 1 s→LUMO_M_ resonances. Theoretical simulation of the spectra, which were computed using the unsupported H‐bonded models of Figure 1, clearly showed that the opposite shifts of the CA/M resonances essentially reflect the shifts undergone by the corresponding N_i_ and N_t_ ionization potentials. Here we investigated the O and C K‐edge spectra of the CA*M assembly in relation to the monomolecular spectra. Figure 2 shows the experimental O (left) and C K‐edge (right) p‐polarized NEXAFS spectra for the mixed sample (CA*M) and the monomolecular samples (CA, M), along with the simulated spectra.

Regarding the experimental O K‐edge spectrum of the CA*M sample, the strong π* resonance (532.6 eV) is assigned to the O 1s transition towards the LUMO π* orbital, localized on the (C−N) and (C=O) bonds of the central CA moiety. It is shifted to higher photon energy (+0.2 eV) compared to the same peak in the monomolecular sample. In the simulation, instead, the O 1s resonances of CA*M practically overlap with those of CA. An explanation of this apparent discrepancy will be given later in the text, after the XPS analysis.

Regarding the C K‐edge spectra, the CA*M curve is characterized by two resonances (288.6 eV, 289.4 eV) which clearly correspond to those of the M (288.6 eV) and CA (289.5 eV) samples. The M transition is towards the LUMO π* (C=N) orbital, localized on the central M moiety of the (CA*M)‐2D model, while the CA transition has a π* (C−N) and (C=O) character. Both experiments and calculations indicate that the energy position of the melamine resonance in CA*M is unchanged with respect to the monomolecular network, while the one associated with the CA molecule shifts to lower energies when passing from CA to CA*M (−0.1 eV in the experiment and −0.25 eV in the simulation).

Despite the good agreement between the experimental and calculated curves, as well as the observed effects of the intermolecular interaction on some spectral features, it is not possible to obtain further details concerning the charge redistribution occurring upon the formation of the CA*M network.

### XPS

To gain a deeper understanding of the chemistry of the CA*M system, we carried out a detailed XPS investigation to elucidate the variations in the O 1s and C 1s IPs values that occur upon the formation of the hydrogen bonded network in the heteromolecular system. We want to recall, here, that the morphology of the mixed sample (co‐presence of mixed and individual domains both in the first and upper layers) does not allow for a parallel analysis of the N 1s XPS spectra. As shown in our previous work,^[12]^ deconvolution of the N 1s spectrum of the mixed sample was indeed nearly impossible due to the high number of components to be used (4 N_i_ components accounting for CA molecules belonging to CA*M and CA domains, both in the first and upper layers; further 8 components accounting for the N_t_ and N_a_ species in CA*M and persisting M domains both in the first and upper layers). On the other hand, O 1s and C 1s XPS spectra of the mixed sample were much easier to analyze due to the presence of fewer components.

**Figure 3:**
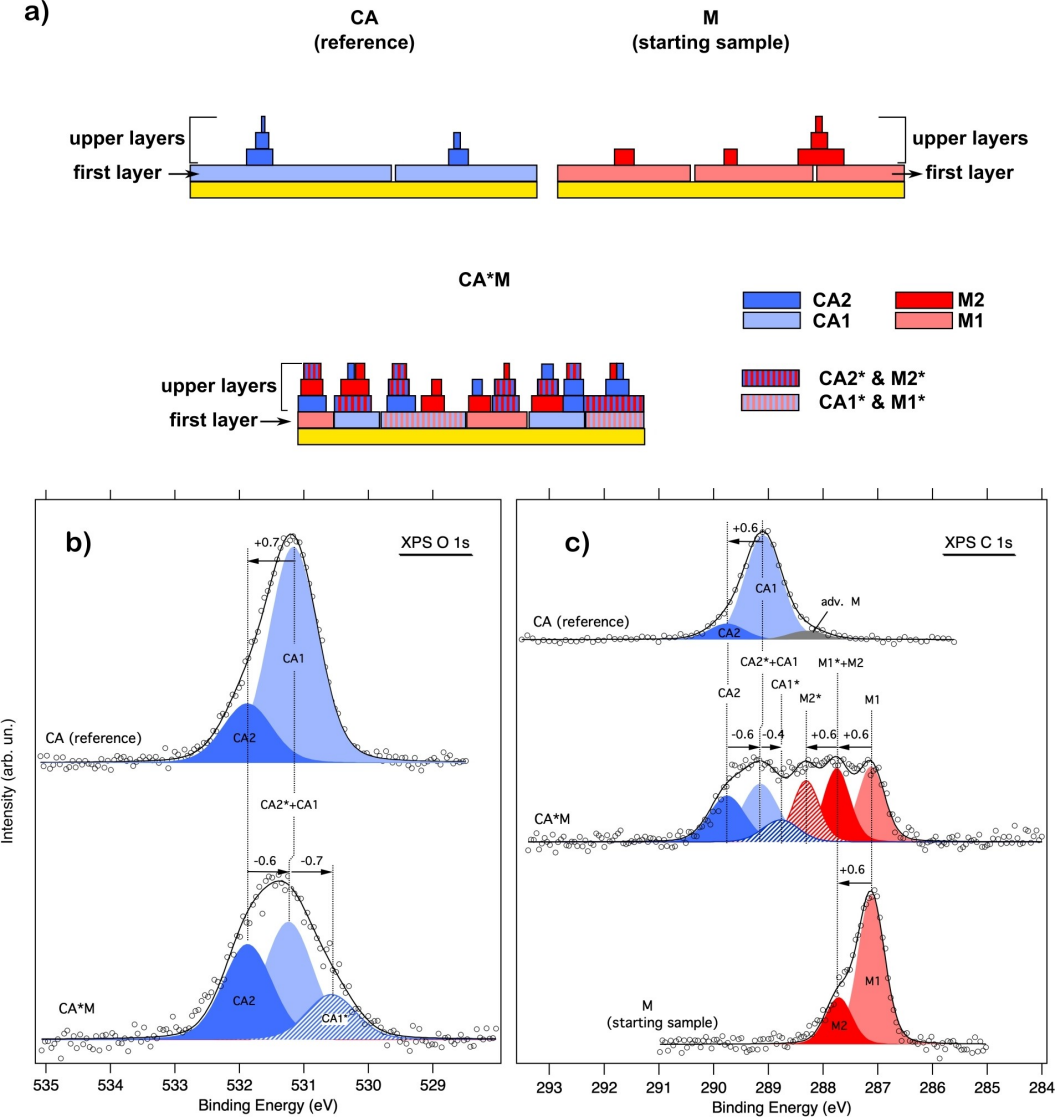
(a) A side view representation of the samples under investigation. (b) XPS O 1s spectra of CA*M and CA samples. (c) XPS C 1s spectra of CA, CA*M and M samples. All spectra are shown with the curve fitting. Circles and black lines represent experimental data and total fit curve, respectively. Blue and red filled curves correspond to CA and M components, respectively.

Figure 3a shows a model representation of the samples under investigation where CA/M molecules within homomolecular domains of the first and upper layers are labeled as CA1/M1 and CA2/M2, respectively. On the other hand, CA1*/M1* and CA2*/M2* stand for CA and M molecules belonging to the mixed domains both in the first and upper layers, respectively. Figure 3b/c shows the O 1s and C 1s photoemission spectra of the CA*M sample along with those of the individual CA/M networks. Both O 1s and C 1s CA spectra are properly described by introducing two main components (labelled CA1 and CA2 respectively), with the less intense one at +0.7 eV (O 1s) and +0.6 eV (C 1s). Similar components were also detected in the corresponding N 1s spectrum (not shown, see previous work^[12]^). As extensively discussed in our previous work,^[12]^ the lower (CA1) and higher (CA2) BE components are likely deriving from first‐ and upper‐layers H‐bonded CA molecules. At variance with the O 1s, the C 1s features an additional small contribution on the lower BE side (gray filled curve), probably due to the adventitious adsorption of melamine (similar features were also detected in the corresponding N 1s spectrum^[12]^).

Like to the CA case, the C 1s XPS spectrum of melamine clearly shows a main peak (M1) at 287.1 eV and a shoulder (M2) at +0.6 eV, which can be attributed to first and multilayer H‐bonded M molecules, respectively. The assignment is further confirmed by the concurrent detection of second layer species in the melamine N 1s spectrum.^[12]^

According to the expected morphology for the mixed sample (co‐presence of CA*M and individual CA/M domains both in the first and upper layers), deconvolution of the O 1s spectrum was initially performed by introducing 4 components: CA1* and CA2* accounting for CA molecules interacting with M molecules respectively in the first and upper layers, and CA1 and CA2 components for first and multilayer CA molecules embedded in segregated homomolecular domains. Interestingly, the fitting procedure leaves only three components: one at 531.8 eV, which clearly corresponds to CA2, and two other peaks at 531.2 eV and 530.5 eV. The peak at 531.2 eV may still stand for CA1 molecules (previously at 531.1 eV), while the third peak at 530.5 eV may correspond to CA1*. This means that the hetero‐interaction shifts the BE of CA by *ca*. −0.7 eV. In absence of a specific molecule‐substrate coupling, both first and upper CA* species should experience a similar negative shift relative to the unreacted species. Hence, the contribution at 531.2 eV, which is at −0.6 eV with respect to CA2, may also arise from CA2*. In the following we will refer to this contribution as (CA2*+CA1).

Following the direction given by the O 1s fitting, the C 1s peak of the mixed sample was fitted by directly introducing three CA components: CA2, (CA2*+CA1) and CA1* (i. e. we did not try to distinguish between CA2* and CA1). Moreover, their intensity ratio was chosen to be equal to that of the O 1s components. Only the BE of CA2 was fixed to the value found for the monomolecular sample. Similarly, three additional components were added for melamine: M1, (M2+M1*) and M2*. In this case, we envisage that M1* and M2* (M molecules interacting with CA in the first and multi‐layer, respectively) will experience a positive shift compared to the corresponding M1 and M2. Hence, M1* will overlap with M2 yielding the (M1*+M2) component while M2* will be shifted to higher BE with respect to (M1*+M2). Only the BE of M1 was kept fixed to the value found in the spectrum of the homomolecular counterpart.

Starting from the CA molecule, the energy position of the CA2*+CA1 component nearly corresponds to that of CA1, hence CA2* is found at −0.6 eV with respect to CA2. On the other hand, CA1* is found at −0.4 eV relative to the corresponding CA1. Regarding the M species, we found that (M1*+M2) component is found at +0.6 eV with respect to M1, very close in energy to M2 of the starting melamine sample. In turn, M2* is found at +0.6 eV relative to (M1*+M2).

O 1 s and C 1s BEs and chemical shifts (*cs*) of the M and CA species corresponding to the fit components obtained through deconvolution of the CA*M spectra are reported in Table 1. Details about the fitting procedure can be found in the Experimental section.

**Table 1:** O 1s and C 1s BEs/cs of the M and CA species corresponding to the fit components obtained through deconvolution of the XPS spectra of the mixed sample. BEs are reported for the M and CA species of the segregated homomolecular domains (both first and upper layers). Chemical shifts (*cs*) are given for M/CA of the hetero‐domains. The experimental values are compared with the O 1s and C 1s IPs/cs computed for the unsupported H‐bonded CA and M molecules of the models reported in Figure 1. N 1s IPs from our previous work^[12]^ are also reported. We want to recall that a direct comparison between experimental BEs and IPs is not possible due to the difference in energy scale calibration. Energies are in eV.

	Exp		IP^ΔKS^	
	CA/M BE	CA*M *cs*	CA/M IP	CA*M *cs*
C (CA)	289.1 (CA1) 289.7 (CA2)	−0.4 (CA1*) −0.6 (CA2*)	295.48	−0.49
C (M)	287.1 (M1) 287.7 (M2)	+0.6 (M1*) +0.6 (M2*)	292.84	+0.42
N_i_ (CA)	–	–	406.12	−0.58
N_t_ (M)	–	–	403.09	+0.49
N_a_ (M)	–	–	404.20	−0.04
O (CA)	531.2 (CA1) 531.8 (CA2)	−0.7 (CA1*) −0.6 (CA2*)	537.10	−0.27

To corroborate the assignment of the components related with the CA*M interaction, we computed C, O and N 1s IPs/*cs* of the central molecules belonging to the unsupported H‐bonded models reported in Figure 1. All values are reported in Table 1 and compared with the experimental counterpart, except for the N 1s level. As shown, all the CA IPs undergo a negative shift when passing from the monomolecular network to the mixed one. On the other hand, positive shifts are observed in the case of the M levels, except for N_a_, whose IP remains almost unchanged. This trend compares well with what found for the experimental fit components, both first and upper layers. Indeed, the C 1s IPs of M and CA shifts, respectively, by +0.4 eV and by −0.5 eV, in very good agreement with the experimental values, +0.6 eV (M) and −0.4/−0.6 eV (CA). Finally, also the O 1s energy level is found to shift to lower values although by a smaller amount (−0.3 eV) compared to the experimental values (−0.7/−0.6 eV).

We can now examine the O 1s NEXAFS spectra with greater insight. As shown both theoretical and experimental results demonstrate that all core levels of the CA molecule shift to lower IPs/BEs values when going from the pure CA sample to the mixed one. Simultaneously, the CA resonances in the C and N K‐edge NEXAFS spectra^[12]^ shift to lower photon energy values. This means that the unoccupied levels involved in these transitions shift in the same direction as the core levels, but with a smaller magnitude. Notably, for the O1s, both theory and experiment reveal that the unoccupied level involved in the NEXAFS transition shifts by a similar amount (theory) or even more (experiment) compared to the core level. Experimentally, this is observed as a shift of the O 1s NEXAFS resonance to higher photon energies, while in theoretical calculations, the energy shifts are slightly underestimated, leading to the NEXAFS peaks being almost perfectly aligned.

### NBO Analysis

**Figure 4:**
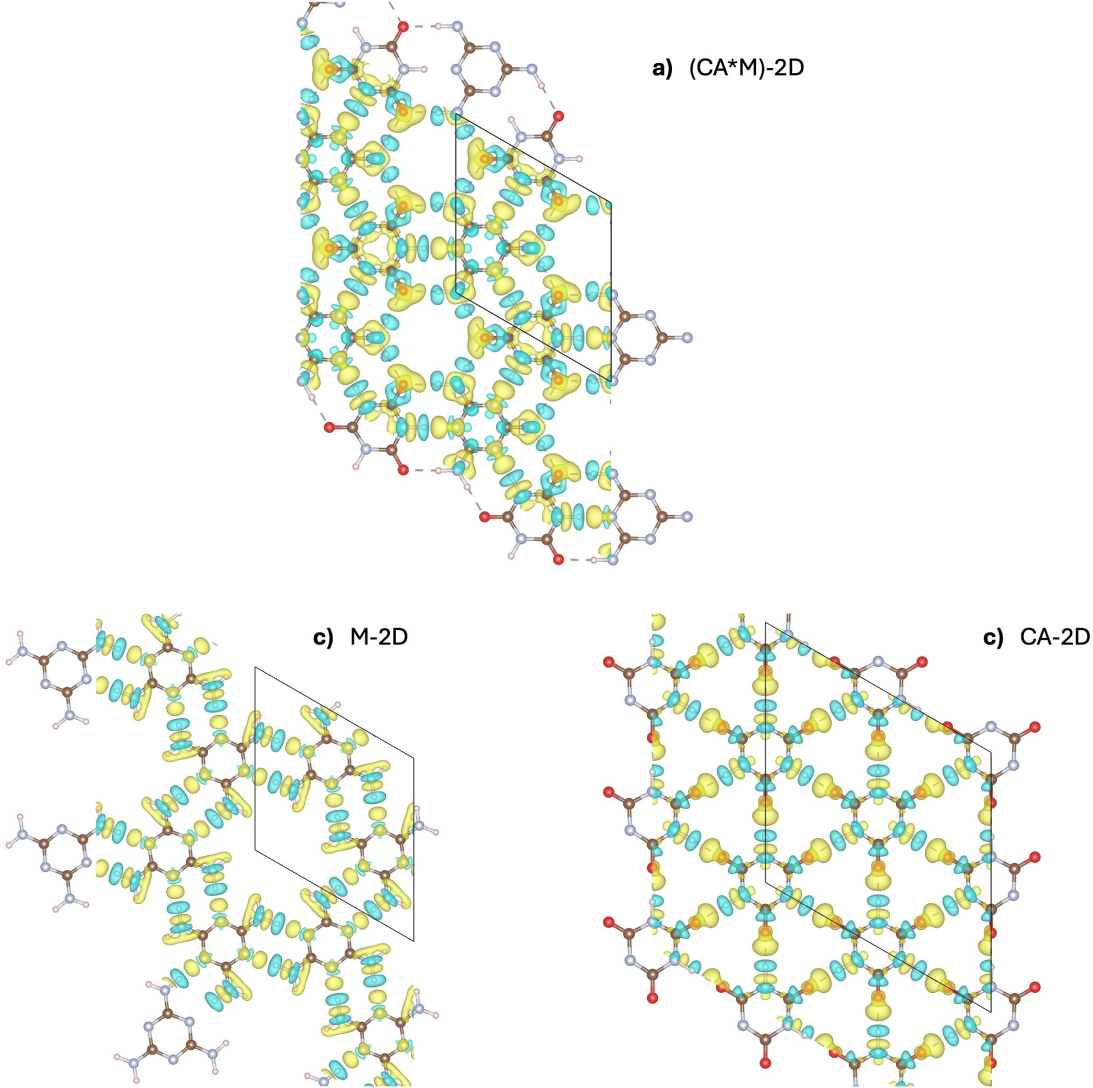
Charge density difference plots obtained for the (a) (CA*M)‐2D (b) M‐2D, (c) CA‐2D H‐bonded models. The electronic charge density redistribution due to the formation of a periodic H‐bonds network is highlighted through yellow/cyan lobes (electron accumulation/depletion). Isosurface value: 0.00186 e^−^/Å^3^.

Upon formation of the hetero‐interaction, all the BEs/IPs of the CA molecule decrease (negative chemical shifts) while those of M increase (positive chemical shifts), suggesting a net charge donation from melamine to cyanuric acid. A qualitative assessment of the charge accumulation/depletion experienced by the two molecules can be made by examining the charge density difference (CDD) plots of the homomolecular and heteromolecular networks (Figure 4), which were computed relative to the isolated molecules. While the homomolecular systems display significant charge redistribution only along the H‐bonding terminations, the CA*M network clearly shows charge accumulation (yellow lobes) within the CA molecule and charge depletion (cyan lobes) within the M unit.

To study in some detail changes in electronic charge distribution of CA and M units that occur upon the formation of the heteromolecular CA*M overlayer we now consider how the stabilization energy 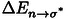 varies for a given set of donor‐acceptor interactions in going from the homomolecular overlayers to the heteromolecular network (in the following it is understood that symbols *n* and *σ* are associated with a given donor Lewis‐type (L) and acceptor non‐Lewis (NL) type NBOs rather than reflecting their symmetry and/or degree of localization on a given atom). Results of such an analysis for the most important interactions are reported in Tables S1‐S6 of the SI while donor‐acceptor NBOs of selected interactions listed in Tables S1‐S2 are reported in Figures S2‐S3. From the relative magnitude of 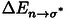 values one could infer that H‐bond strength is overall comparable for homomolecular M and CA overlayers, and slightly stronger for the former. The strength of the 
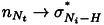
 heteromolecular H‐bond interaction is roughly twice than that corresponding to the 
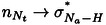
 and 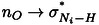 which characterize the intermolecular H‐bond interaction in M and CA overlayers respectively.

Moreover, the strength of the N_i_−H⋅⋅N_t_ interaction exceeds the one associated with the N_a_−H⋅⋅O bond, suggesting a net charge transfer from M to CA upon the formation of the CA*M assembly. This conclusion is supported by a natural population analysis performed on the heteromolecular overlayer, which shows an excess negative charge of about −0.15 e^−^ on unit 4 (CA) and a positive charge of +0.14 e^−^ on unit 2 (M) (see Figure 1). A discussion on the comparison of the evolution of N(i,a)−H bond distances in going from the isolated monomers to the homo‐ and hetero‐molecular structures that arise as a result of the donor‐acceptor H‐bonding interaction, and which is in line with the findings above, is reported in the SI.

**Figure 5:**
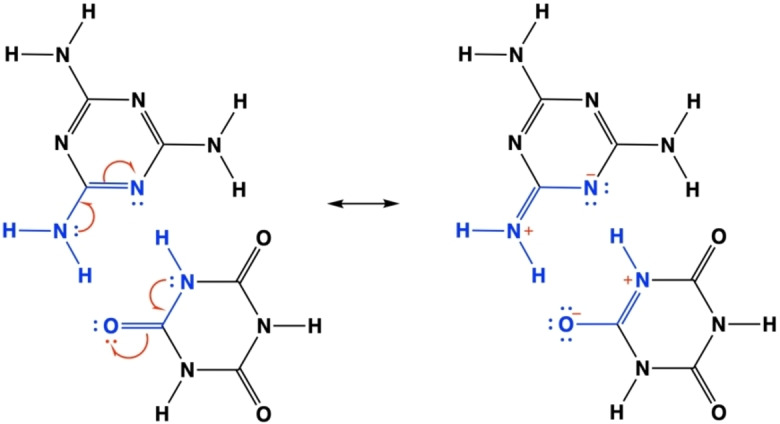
Resonance structure for a portion of the CA*M network.

Finally, one should notice (see Tables S1–S6) that the *intramolecular* interactions (electron delocalization) involving the amino N lone pair (
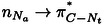
 ) and the imide N lone pair (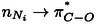 ) are stabilized upon the formation of the extended H‐bond networks in the homomolecular overlayers. An extra‐stabilization of *intra*‐molecular delocalization effects is further apparent because of the formation of the heteromolecular overlayer. These results indicate an interplay between hydrogen bonds and mesomeric effects, both playing a role in the charge redistributions within the two molecules. The reinforcement of the mesomeric effects in the CA*M network can be also appreciated by comparing the CDD plots of Figure 4. In the (CA*M)‐2D system, we clearly observe cyan lobes (charge depletion) on top of the N_a_ and yellow lobes (charge accumulation) on the N_t_, along with some charge depletion inside the ring, all of which in line with the 
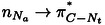
 interaction. For the CA molecule, instead, we clearly observe a charge depletion on top the N_i_ and charge accumulation inside the ring, again in line with the 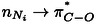 interaction. Both interactions are usually described by considering the resonance structures shown in Figure 5.

## Conclusions

In conclusion, we investigated how the electronic properties of the CA and M molecules are affected by the H‐bonding interactions existing in the CA*M adduct, i. e. N_i_−H⋅⋅N_t_ and N_a_−H⋅⋅O. The study was conducted by comparing XPS and NEXAFS spectra of the monomolecular H‐bonded assemblies with those of the bimolecular sample, all grown on the Au(111) surface. Specifically, it was found that the O 1s and C 1s XPS spectra of the mixed sample can be described by the superposition of the individual molecular spectra, along with the addition of new components at lower BEs components for CA and higher BEs for M. These findings indicate that the mixed sample is characterized by the coexistence of monomolecular and bimolecular domains, with the latter responsible for the new components. The core level shifts undergone by M and CA upon formation of the hetero interaction were further confirmed by theoretical simulation of the IPs, which were computed by using unsupported models of the three systems.

Rationalization of the observed BEs/IPs shifts was then obtained through NBO analysis of both *intermolecular* (H‐bonds) and *intramolecular* interactions (electron delocalization effects) within the three networks. The analysis reveals that the strength of the N_i_−H⋅⋅N_t_ hydrogen bond is significantly greater than the N_a_−H⋅⋅O interaction, which is, in turn, comparable to or weaker than the homomolecular ones. Consequently, the charge donation from M to CA *via* N_i_−H⋅⋅N_t_ is more important to the back‐donation from CA to M *via* N_a_−H⋅⋅O, thereby justifying the sign of the core level shifts. Finally, the NBO analysis reveals a gradual strengthening of the electron delocalization effects when going form the isolated molecule to the monomolecular and heteromolecular networks. This indicates an interplay between H‐bonds and electron delocalization effects, both of which contribute to charge redistribution within the two molecules.

## Conflict of Interests

The authors declare no conflict of interest.

1

## Supporting information

As a service to our authors and readers, this journal provides supporting information supplied by the authors. Such materials are peer reviewed and may be re‐organized for online delivery, but are not copy‐edited or typeset. Technical support issues arising from supporting information (other than missing files) should be addressed to the authors.

Supporting Information

## Data Availability

The data that support the findings of this study are available from the corresponding author upon reasonable request.

## References

[1] J. P. Mathias , E. E. Simanek , J. A. Zerkowski , C. T. Seto , G. M. Whitesides , J. Am. Chem. Soc. 1994, 116(10), 4316–4325. 10.1021/ja00089a021.

[2] J. A. Zerkowski , J. C. MacDonald , C. T. Seto , D. A. Wierda , G. M. Whitesides , J. Am. Chem. Soc. 1994, 116(6), 2382–2391. 10.1021/ja00085a018.

[3] J. A. Zerkowski , C. T. Seto , D. A. Wierda , G. M. Whitesides , J. Am. Chem. Soc. 1990, 112, 9025–9026. 10.1021/ja00180a083.

[4] D. C. Sherrington , K. A. Taskinen , Chem. Soc. Rev. 2001, 30(2), 83–93. 10.1039/b008033k.

[5] Y. Li , J. Xu , C. Sun , RSC Adv. 2015, 5(2), 1125–1147. 10.1039/c4ra13080d.

[6] K. Ai , Y. Liu , L. Lu , J. Am. Chem. Soc. 2009, 131(27), 9496–9497. 10.1021/ja9037017. 19537721

[7] T. Sanji , M. Nakamura , S. Kawamata , M. Tanaka , S. Itagaki , T. Gunji , Chem. Eur. J. 2012, 18(48), 15254–15257. 10.1002/chem.201203081. 23129529

[8] V. V. Korolkov , M. Baldoni , K. Watanabe , T. Taniguchi , E. Besley , P. H. Beton , Nat. Chem. 2017, 9(12), 1191–1197. 10.1038/nchem.2824. 29168478

[9] Z. Liu , K. Sun , X. Li , L. Li , H. Zhnag , J. Phys. Chem. Lett. 2019, 10, 4297–4302. 10.1021/acs.jpclett.9b01167. 31318568

[10] C. Wang , R. Furlan de Oliveira , K. Jiang , Y. Zhao , N. Turetta , C. Ma , B. Han , H. Zhang , D. Tranca , X. Zhuang , L. Chi , A. Ciesielski , P. Samorì , Nat. Commun. 2022, 13(1). 10.1038/S41467-022-28116-Y. PMC8791956 35082288

[11] J. Wu , P. Bag , Y. Xu , L. Gong , C. He , X. Chen , Adv. Mater. 2021, 33, 2007368. 10.1002/adma.202007368. 33893666

[12] G. Fronzoni , A. Cossaro , R. Costantini , L. Di Nardi , E. Viola , G. Balducci , V. Lanzilotto , D. Toffoli , Appl. Surf. Sci. 2024, 642, 158573. 10.1016/j.apsusc.2023.158573.

[13] K. Damodaran , G. J. Sanjayan , P. R. Rajamohanan , S. Ganapathy , K. N. Ganesh , Org. Lett. 2001, 3(12), 1921–1924. 10.1021/ol0159672. 11405745

[14] A. N. Petelski , D. J. R. Duarte , S. C. Pamies , N. M. Peruchena , G. L. Sosa , Theor. Chem. Acc. 2016, 135(3), 1–13. 10.1007/s00214-015-1795-3.

[15] R. Costantini , M. Stredansky , D. Cvetko , G. Kladnik , A. Verdini , P. Sigalotti , F. Cilento , F. Salvador , A. De Luisa , D. Benedetti , L. Floreano , A. Morgante , A. Cossaro , M. Dell'Angela , J. Electron Spectrosc. Relat. Phenom. 2018, 229, 7–12. 10.1016/j.elspec.2018.09.005.

[16] W. Xu , M. Dong , H. Gersen , E. Rauls , S. Vázquez-Campos , M. Crego-Calama , D. N. Reinhoudt , I. Stensgaard , E. Laegsgaard , T. R. Linderoth , F. Besenbacher , Small 2007, 3(5), 854–858. 10.1002/smll.200600407. 17393548

[17] C. Nicolas , J. Electron Spectrosc. Relat. Phenom. 2012, 185(8–9), 267–272. 10.1016/j.elspec.2012.05.008.

[18] P. Giannozzi , S. Baroni , N. Bonini , M. Calandra , R. Car , C. Cavazzoni , D. Ceresoli , G. L. Chiarotti , M. Cococcioni , I. Dabo , A. Dal Corso , S. de Gironcoli , S. Fabris , G. Fratesi , R. Gebauer , U. Gerstmann , C. Gougoussis , A. Kokalj , M. Lazzeri , L. Martin-Samos , N. Marzari , F. Mauri , R. Mazzarello , S. Paolini , A. Pasquarello , L. Paulatto , C. Sbraccia , S. Scandolo , G. Sclauzero , A. P. Seitsonen , A. Smogunov , P. Umari , R. M. Wentzcovitch , J. Phys. Condens. Matter 2009, 21(39), 395502–395520. 10.1088/0953-8984/21/39/395502. 21832390

[19] V. Lanzilotto , D. Toffoli , E. Bernes , M. Stener , E. Viola , A. Cossaro , R. Costantini , C. Grazioli , R. Totani , G. Fronzoni , J. Phys. Chem. A 2022, 126(39), 6870–6881. 10.1021/acs.jpca.2c04517. 36168982 PMC9549465

[20] J. C. Slater , Adv. Quantum Chem. 1972, 6(C), 1–92. 10.1016/S0065-3276(08)60541-9.

[21] L. Triguero , L. G. M. Pettersson , H. Ågren , Phys. Rev. B 1998, 58 (12), 8097–8110. 10.1103/PhysRevB.58.8097.

[22] A. D. Becke , J. Chem. Phys. 1993, 98(7), 5648–5652. 10.1063/1.464913.

[23] C. Lee , W. Yang , R. G. Parr , Phys. Rev. B 1988, 37(2), 785. 10.1103/PhysRevB.37.785. 9944570

[24] P. J. Stephens , F. J. Devlin , C. F. Chabalowski , M. J. Frisch , J. Phys. Chem. 1994, 98(45), 11623–11627. 10.1021/j100096a001.

[25] G. Te Velde , F. M. Bickelhaupt , E. J. Baerends , C. Fonseca Guerra , S. J. A. van Gisbergen , J. G. Snijders , T. Ziegler , J. Comput. Chem. 2001, 22(9), 931–967. 10.1002/JCC.1056.

[26] C. Fonseca Guerra , J. G. Snijders , G. Te Velde , E. J. Baerends , Theor. Chem. Acc. 1998, 99(6), 391–403. 10.1007/S002140050353.

[27] ADF 2023.1, SCM, Theoretical Chemistry. Vrije Universiteit: Amsterdam, The Netherlands. https://www.scm.com.

[28] J. P. Foster , F. Weinhold , J. Am. Chem. Soc. 1980, 102(24), 7211–7218. 10.1021/JA00544A007.

[29] A. E. Reed , F. Weinhold , J. Chem. Phys. 1985, 83(4), 1736–1740. 10.1063/1.449360.

[30] F. Weinhold, *Natural Bond Orbital Methods*; Ed. P. von Ragué Schleyer, Wiley: Chichester, United Kingdom, 1998.

[31] F. Weinhold , R. A. Klein , Mol. Phys. 2012, 110(9–10), 565–579. 10.1080/00268976.2012.661478.

[32] F. Weinhold , C. R. Landis , E. D. Glendening , Int. Rev. Phys. Chem. 2016, 35(3), 399–440. 10.1080/0144235X.2016.1192262.

[33] E. D. Glendening , C. R. Landis , F. Weinhold , Wiley Interdiscip. Rev.: Comput. Mol. Sci. 2012, 2(1), 1–42. 10.1002/WCMS.51.

