# Supplementary material for: Inter‐ and Intra‐Molecular Charge Redistributions in H‐Bonded Cyanuric Acid*Melamine (CA*M) Networks: Insight From Core Level Spectroscopy and Natural Bond Orbital Analysis
Source: Chemistry. 2025 Jan 7;31(11):e202403782. doi: 10.1002/chem.202403782 (PMC11840656; doi:10.1002/chem.202403782)
Supplement: Supplementary file 1 — Supporting Information [file CHEM-31-e202403782-s001.pdf]

Chemistry–A European Journal

Supporting Information

Inter- and *Intra*-Molecular Charge Redistributions in H-Bonded Cyanuric Acid*Melamine (CA*M) Networks: Insight From Core Level Spectroscopy and Natural Bond Orbital Analysis

Daniele Toffoli,* Roberto Costantini, Elisa Bernes, Lorys Di Nardi, Gabriele Balducci, Elisa Viola, Giovanna Fronzoni, Albano Cossaro, and Valeria Lanzilotto*

Supporting Material for: Inter- and *intra*-molecular charge redistributions in H-bonded Cyanuric Acid*Melamine (CA*M) networks: insight from core level spectroscopy and natural bond orbital analysis

Daniele Toffoli,[a,b,*] Roberto Costantini,[c,b] Elisa Bernes,[a] Gabriele Balducci,[a] Elisa Viola,[d] Lorys Di Nardi,[d] Giovanna Fronzoni,[a] Albano Cossaro,[a,b] Valeria Lanzilotto[a,b,d*]

[a]*Dipartimento di Scienze Chimiche e Farmaceutiche, Università degli Studi di Trieste, 34127, Trieste, Italy*

[b]*IOM-CNR, Istituto Officina dei Materiali-CNR, S.S.14, Km 163.5, 34149 Trieste, Italy*

[c]*Dipartimento di Fisica, Università degli Studi di Trieste, 34127, Trieste, Italy*

[d]*Dipartimento di Chimica, Sapienza Univeristà di Roma, 00185, Italy*

*Corresponding authors: toffoli@units.it, valeria.lanzilotto@units.it

S0. Growth of the homomolecular and the heteromolecular samples

The growth of the homomolecular and mixed films was monitored by acquiring *fast* XPS scans during molecules deposition. In the case of the CA sample, we monitored both O 1s and Au $4f_{7/2}$ levels by using a photon energy of 650 eV. As for the M and CA*M samples we looked at the N 1s and Au Au $4f_{7/2}$ levels with $h\nu$ = 515 eV. The spectra were acquired with emission angle (θ) of 50° and grazing incidence (GI). Such a geometry allowed for simultaneous acquisition of the spectra and molecule deposition. NB: the spectra reported in the main text are acquired with θ = 35° and normal incidence (NI). Then, we obtained the Au4f/O1s (CA) and Au4f/N1s (M, CA*M) ratios using the areas underneath the photoemission peaks corrected for the corresponding cross sections.

Figure S0a shows the evolution of the Au4f/O1s ratio while depositing the CA molecule to obtain the CA "reference" sample. Figure S0b shows the evolution of the Au4f/N1s ratio while depositing the M molecule for obtaining the M sample, the so-called "starting sample". Figure S0c reports the evolution of the Au4f/N1s ratio during the formation of the CA*M sample, which was prepared by depositing the CA molecule on top the M "starting sample" (the latter obtained after 10' of M deposition).

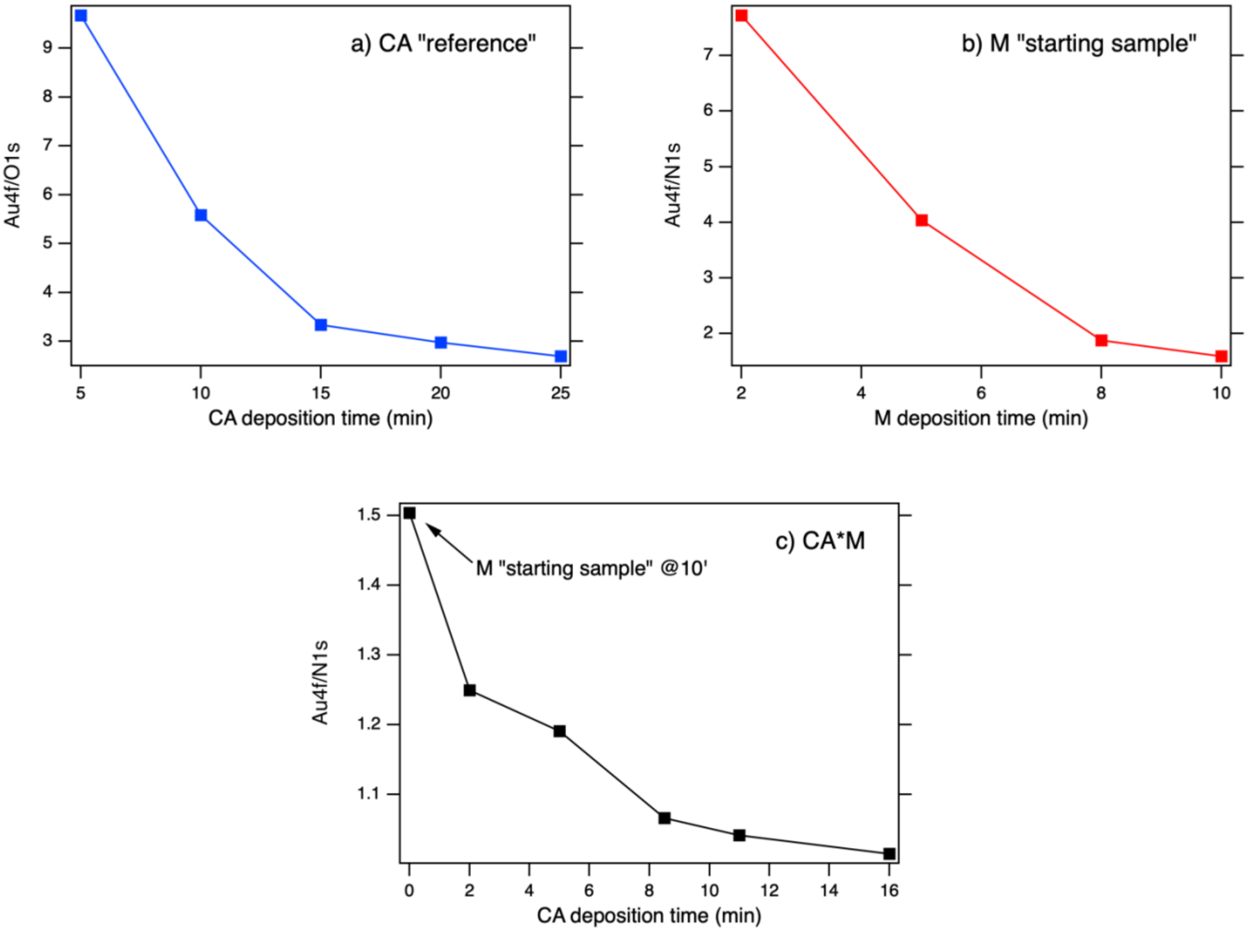

Figure S0. Evolution of the Au4f/O1s ratio during formation of the CA "reference" sample (a). Evolution of the Au4f/N1s ratio during the formation of the M "starting sample" (b) and the CA*M sample (c). The M sample obtained after 10' deposition of the M molecules was then used for the deposition of the CA molecule to grow the mixed film.

As shown, the growth of the homomolecular films is characterized by the occurrence of a saturation coverage, i.e. both Au4f/O1s and Au4f/N1s reach a kind of plateau. The occurrence of a saturation coverage is a sign that the metal surface is completely covered, and further molecules do not easily stick on top the adsorbed molecular layer(s). Deconvolution of the O1s/N1s spectra reveals the presence of two components

since the early stages of both depositions. Such features can be interpreted as the occurrence of a Stransky-Krastanov growth, where the main component at lower BEs (CA1, M1) can be attributed to molecules in the 1st layer and the smaller component at higher BEs to upper layers molecules. According to the relative intensity between the two components and considering attenuation effects of the 1st layer signals, we estimate a nominal coverage of ~1.2 ML for CA and ~1.4 ML for melamine (see the O 1s and C1s spectra shown in the main text and the N 1s spectra in our previous publication ref. 1).

As already mentioned, the M sample obtained after 10' of deposition was used as playground for the CA deposition to obtain the CA*M sample. While further M molecules do not easily stick on top the 10' M sample, the adsorption of CA molecules is rather effective. The heteromolecular H-bonding interaction promotes the intercalation of CA molecules within the pre-formed M domains. The presence of both CA and M molecules in upper layers states that also the mixed system is characterized by a Stransky-Krastanov morphology.

It must be noticed that the mixed sample was prepared by dosing CA molecules for 16', which is the same time needed to reach the saturation coverage for the CA reference sample. Hence, we can infer to have dosed at least the equivalent of ~1.2 ML of pure CA domains.

S1. NBO analysis of CA*M H-bonding interaction

In Figure S1 we report a scheme of the H-bonding network considered in the NBO analysis with a numbering of the different subunits (1-6). In table S1 and S2 we report the second-order perturbation theory analysis for units 2 and 4. In the tables, along with the analysis of the intermolecular H-bond interactions with the neighboring units we also report donor-acceptor delocalizations within each unit, which underlines intramolecular electron delocalization effects. sp^{λ} valence hybrid orbitals (NHOs) on atom A, reported in the Tables are defined as:

$$h_A = (1+\lambda)^{-1/2}[s_A + \sqrt{\lambda} p_A] \qquad \text{(S1)}$$

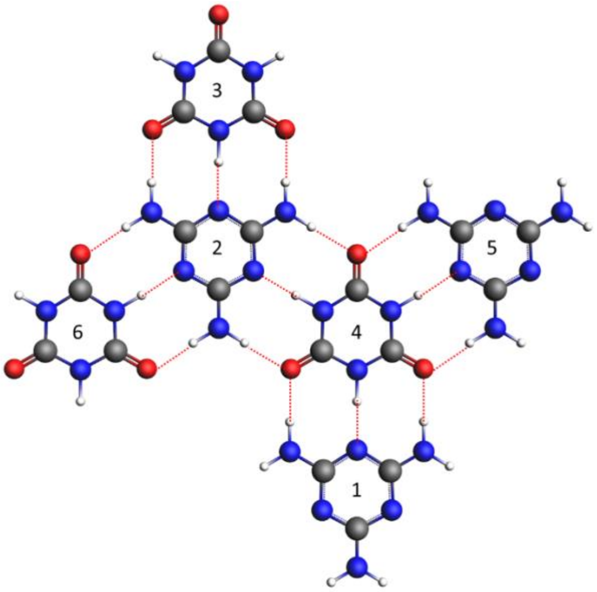

Figure S1. Intermolecular H-bonding network resulting from the CA*M interaction in the overlayer. Also reported is the numbering of the individual CA and M units which are used in the analysis of the NBO donor-acceptor model of H-bonding reported in Tables S1 and S2.

Table S1. Second order perturbation theory analysis of KS matrix in the NBO basis for unit 2 (M, see Figure S1). For NBOs of lone-pair nature, the natural hybrid orbital (NHO) composition is also reported.

donor NBO (unit)	occ.	acceptor NBO (unit)	occ.	$\Delta E_{n\rightarrow\sigma^*}$ (kcal/mol)
H-bond intermolecular interactions				
$n_{N_t}(sp^{1.80}$,2)	1.81803	$\sigma^*_{N_i-H}$ (3)	0.11711	46.40
n_{N_t} ($sp^{1.80}$,2)	1.82076	$\sigma^*_{N_i-H}$ (4)	0.11546	45.90
n_{N_t} ($sp^{1.80}$,2)	1.81855	$\sigma^*_{N_i-H}$ (6)	0.11653	46.23
n_O (p,3)	1.85249	$\sigma^*_{N_a-H}$ (2)	0.03808	12.02
n_O ($sp^{0.55}$,3)	1.97128	$\sigma^*_{N_a-H}$ (2)	0.03888	4.75
n_O ($sp^{0.55}$,3)	1.97132	$\sigma^*_{N_a-H}$ (2)	0.03886	4.75
n_O (p,3)	1.85212	$\sigma^*_{N_a-H}$ (2)	0.03886	12.42
n_O ($sp^{0.53}$,4)	1.95629	$\sigma^*_{N_a-H}$ (2)	0.03691	6.34
n_O ($sp^{0.53}$,4)	1.95652	$\sigma^*_{N_a-H}$ (2)	0.03750	6.30
n_O (p,4)	1.84753	$\sigma^*_{N_a-H}$ (2)	0.03691	9.51
n_O (p,4)	1.84792	$\sigma^*_{N_a-H}$ (2)	0.03750	9.84
n_O ($sp^{0.55}$, 6)	1.97104	$\sigma^*_{N_a-H}$ (2)	0.03932	4.90
n_O ($sp^{0.55}$, 6)	1.97105	$\sigma^*_{N_a-H}$ (2)	0.03876	4.92
n_O (p,6)	1.85155	$\sigma^*_{N_a-H}$ (2)	0.03932	12.60
n_O (p,6)	1.85249	$\sigma^*_{N_a-H}$ (2)	0.03876	12.34
intramolecular donor-acceptor interactions				
$n_{N_a}(p)$	1.68297	$\pi^*_{C-N_t}$	0.52627	86.41
$n_{N_t}(sp^{1.80})$	1.81803	$\sigma^*_{N_t-C}$	0.03716	11.26
$n_{N_t}(sp^{1.80})$	1.81803	$\sigma^*_{N_t-C}$	0.03727	11.29
$n_{N_a}(p)$	1.68351	$\pi^*_{C-N_t}$	0.52520	86.06
$n_{N_a}(p)$	1.68022	$\pi^*_{C-N_t}$	0.52740	87.09
$n_{N_t}(sp^{1.80})$	1.82076	$\sigma^*_{N_t-C}$	0.03716	11.31
$n_{N_t}(sp^{1.80})$	1.82076	$\sigma^*_{N_t-C}$	0.03727	11.30
$n_{N_t}(sp^{1.80})$	1.81855	$\sigma^*_{N_t-C}$	0.03733	11.29
$n_{N_t}(sp^{1.80})$	1.81855	$\sigma^*_{N_t-C}$	0.03719	11.27
π_{C-N_t}	1.77316	$\pi^*_{C-N_t}$	0.52520	50.54
π_{C-N_t}	1.77276	$\pi^*_{C-N_t}$	0.52627	50.64
π_{C-N_t}	1.77387	$\pi^*_{C-N_t}$	0.52740	50.59

Table S2. Second order perturbation theory analysis of KS matrix in the NBO basis for unit 4 (CA, see Figure S1). For NBOs of lone-pair nature, the natural hybrid orbital (NHO) composition is also reported.

donor NBO (unit)	occ.	acceptor NBO (unit)	occ.	$\Delta E_{n\rightarrow\sigma^*}$ (kcal/mol)
H-bond intermolecular interactions				
$n_{N_t}(sp^{1.85}$,1)	1.81931	$\sigma^*_{N_i-H}$ (4)	0.12016	47.20
n_{N_t} $(sp^{1.80}$,2)	1.82076	$\sigma^*_{N_i-H}$ (4)	0.11546	45.90
n_{N_t} $(sp^{1.85}, 5)$	1.81985	$\sigma^*_{N_i-H}$ (4)	0.11957	47.03
n_O $(sp^{0.53}$,4)	1.95629	$\sigma^*_{N_a-H}$ (5)	0.03810	6.45
n_O $(sp^{0.53}$,4)	1.95623	$\sigma^*_{N_a-H}$ (5)	0.03768	6.51
n_O (p,4)	1.84753	$\sigma^*_{N_a-H}$ (5)	0.03810	10.06
n_O (p,4)	1.84769	$\sigma^*_{N_a-H}$ (5)	0.03768	9.77
n_O $(sp^{0.53}$,4)	1.95652	$\sigma^*_{N_a-H}$ (1)	0.03690	6.37
n_O $(sp^{0.53}$,4)	1.95623	$\sigma^*_{N_a-H}$ (1)	0.03776	6.31
n_O (p,4)	1.84769	$\sigma^*_{N_a-H}$ (1)	0.03776	9.88
n_O (p,4)	1.84792	$\sigma^*_{N_a-H}$ (1)	0.03690	9.50
n_O $(sp^{0.53}$, 4)	1.95629	$\sigma^*_{N_a-H}$ (2)	0.03691	6.34
n_O (p,4)	1.84753	$\sigma^*_{N_a-H}$ (2)	0.03691	9.51
n_O (p,4)	1.84792	$\sigma^*_{N_a-H}$ (2)	0.03750	9.84
intramolecular donor-acceptor interactions				
$n_{N_i}(p)$	1.60046	π^*_{C-O}	0.39456	72.33
$n_{N_i}(p)$	1.60046	π^*_{C-O}	0.39681	72.65
$n_{N_i}(p)$	1.60052	π^*_{C-O}	0.39681	72.67
$n_{N_i}(p)$	1.60052	π^*_{C-O}	0.39559	72.52
$n_{N_i}(p)$	1.60312	π^*_{C-O}	0.39456	71.85
$n_{N_i}(p)$	1.60312	π^*_{C-O}	0.39559	72.09
$n_O(p)$	1.84753	$\sigma^*_{N_i-C}$	0.06913	22.50
$n_O(p)$	1.84753	$\sigma^*_{N_i-C}$	0.06924	22.64
$n_O(p)$	1.84792	$\sigma^*_{N_i-C}$	0.06906	22.60
$n_O(p)$	1.84792	$\sigma^*_{N_i-C}$	0.06932	22.58
$n_O(p)$	1.84769	$\sigma^*_{N_i-C}$	0.06905	22.52
$n_O(p)$	1.84769	$\sigma^*_{N_i-C}$	0.06903	22.50
n_O $(sp^{0.53})$	1.95629	ryd.(C vicinal)	0.00918	13.42
n_O $(sp^{0.53})$	1.95652	ryd.(C vicinal)	0.00921	13.45
n_O $(sp^{0.53})$	1.95623	ryd.(C vicinal)	0.00920	13.30
σ_{N_i-H}	1.97406	$\sigma^*_{N_i-C}$	0.06903	4.16
σ_{N_i-H}	1.97406	$\sigma^*_{N_i-C}$	0.06932	4.20
σ_{N_i-H}	1.97405	$\sigma^*_{N_i-C}$	0.06905	4.18
σ_{N_i-H}	1.97405	$\sigma^*_{N_i-C}$	0.06924	4.18

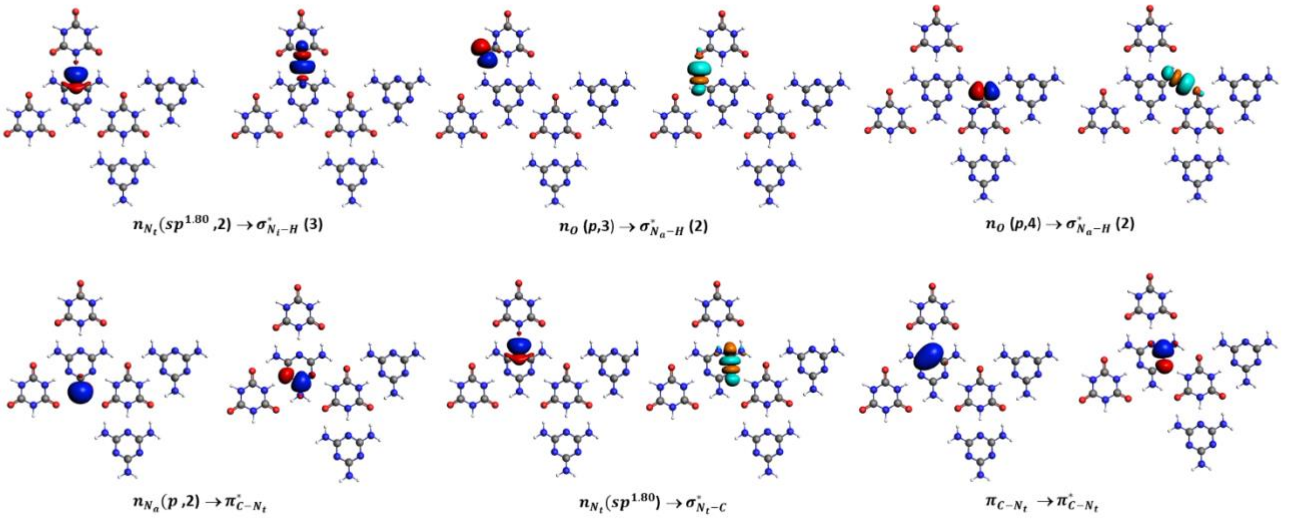

Figure S2. Donor-acceptor NBOs of selected interactions reported in Table S1. Upper row: H-bond intermolecular interactions. Bottom row: intramolecular interactions.

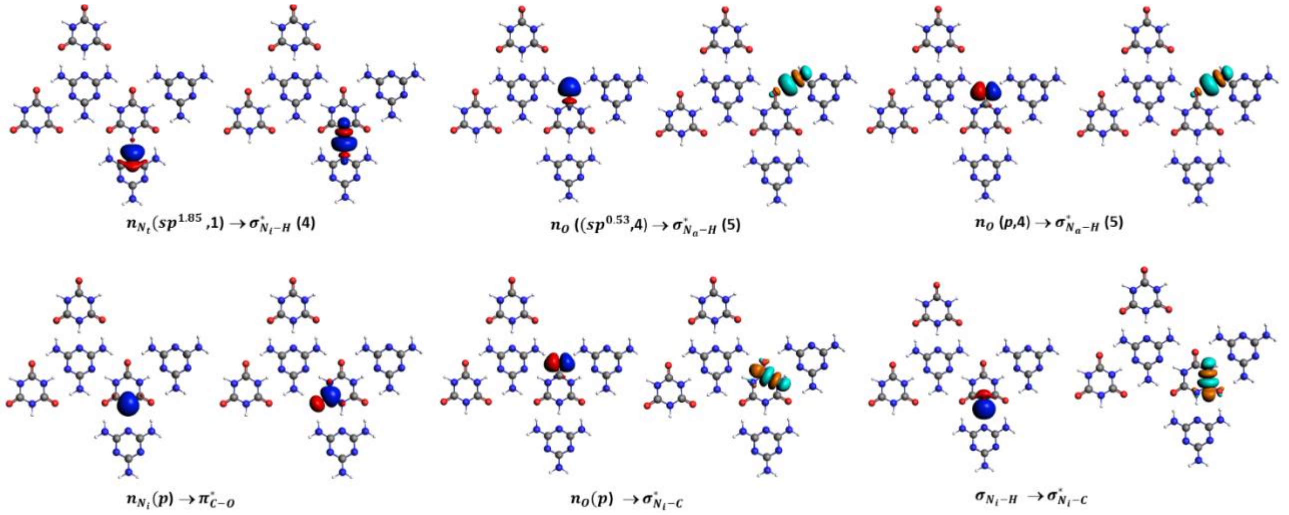

Figure S3. Donor-acceptor NBOs of selected interactions reported in Table S2. Upper row: H-bond intermolecular interactions. Bottom row: intramolecular interactions.

The donor-acceptor interactions reported in Tables S1-S2 are indication of both supramolecular aggregation induced by the H-bond formation, and of conjugation within each units (either CA or M). In a standard NBO analysis this is reflected in departures from the ideal natural Lewis structure (NLS) due to charge transfer from occupied Lewis-type NBOs to unoccupied non-Lewis (NL) NBOs. In fact, for the supramolecular aggregate reported in Fig. S1, the best NLS is unable to account for as much as 3% of the total valence charge (which is reflected in occupations which sometimes strongly deviate from 2.0 in L-type NBOs, see Tables S1-S2). As concerns resonance-type delocalization effects, the delocalization (arrow-pushing) is compatible with N_a-C (unit 2) and N_t-C (unit 4) π bond character). Finally, a natural population analysis revealed an excess charge of about -0.15 e on unit 4 (CA) and a positive charge of +0.14 e on unit 2 (M).

To investigate whether heteromolecular H-bond formation stabilizes intramolecular delocalization, the second order perturbation theory analysis of KS matrix in the NBO basis for the 2D M and CA overlayers is reported in Tables S3 and S4 respectively. Moreover, the same analysis for isolated M and CA is reported in

Table S5 and S6 below. We refer to Fig. S4 for the adopted numbering scheme used in the case of homomolecular overlayers.

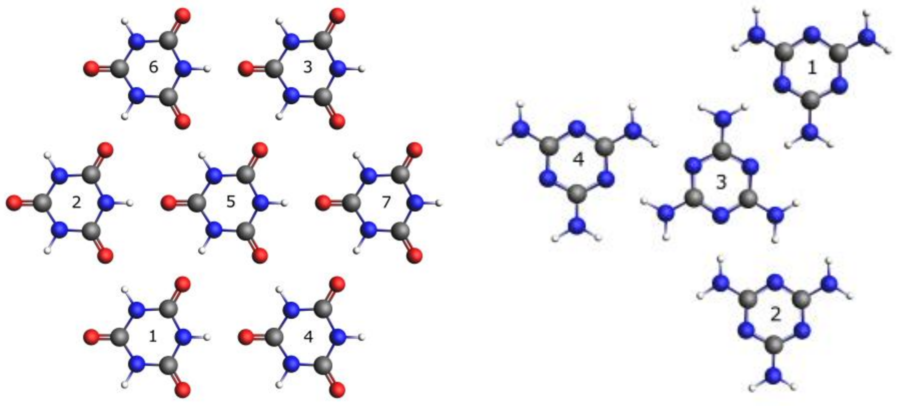

Figure S4. Left panel: Homomolecular CA 2D overlayer; right panel: homomolecular M 2D overlayer. Also reported is the numbering of the individual CA and M units which are used in the analysis of the NBO donor-acceptor model of H-bonding reported in Tables S3-S4.

Table S3. Second order perturbation theory analysis of KS matrix in the NBO basis for unit 5 (CA-2D, see Figure S4). For NBOs of lone-pair nature, the natural hybrid orbital (NHO) composition is also reported.

donor NBO (unit)	occ.	acceptor NBO (unit)	occ.	$\Delta E_{n\rightarrow\sigma^*}$ (kcal/mol)
H-bond intermolecular interactions				
n_O $(sp^{0.58}, 5)$	1.94134	$\sigma^*_{N_i-H}$ (2)	0.04656	21.92
n_O $(sp^{0.58}, 5)$	1.94314	$\sigma^*_{N_i-H}$ (3)	0.04656	21.92
n_O $(sp^{0.58}, 5)$	1.94314	$\sigma^*_{N_i-H}$ (4)	0.04656	21.92
n_O $(sp^{0.58}, 1)$	1.94345	$\sigma^*_{N_i-H}$ (5)	0.04660	21.92
n_O $(sp^{0.58}, 6)$	1.94345	$\sigma^*_{N_i-H}$ (5)	0.04661	21.92
n_O $(sp^{0.58}, 7)$	1.94345	$\sigma^*_{N_i-H}$ (5)	0.04661	21.92
intramolecular donor-acceptor interactions				
$n_{N_i}(p)$	1.62331	π^*_{C-O}	0.37367	66.69
$n_{N_i}(p)$	1.62331	π^*_{C-O}	0.37367	66.69
$n_{N_i}(p)$	1.62331	π^*_{C-O}	0.37367	66.69
$n_{N_i}(p)$	1.62331	π^*_{C-O}	0.37367	66.69
$n_{N_i}(p)$	1.62331	π^*_{C-O}	0.37367	66.69
$n_{N_i}(p)$	1.62331	π^*_{C-O}	0.37367	66.69
$n_O(p)$	1.86604	$\sigma^*_{N_i-C}$	0.07604	23.94
$n_O(p)$	1.86604	$\sigma^*_{N_i-C}$	0.07604	23.94
$n_O(p)$	1.86604	$\sigma^*_{N_i-C}$	0.07604	23.94
$n_O(p)$	1.86604	$\sigma^*_{N_i-C}$	0.07604	23.94
$n_O(p)$	1.86604	$\sigma^*_{N_i-C}$	0.07604	23.94
$n_O(p)$	1.86604	$\sigma^*_{N_i-C}$	0.07604	23.94
n_O $(sp^{0.58})$	1.94314	ryd.(C vicinal)	0.00996	15.48
n_O $(sp^{0.58})$	1.94314	ryd.(C vicinal)	0.00996	15.48
n_O $(sp^{0.58})$	1.94314	ryd.(C vicinal)	0.00996	15.48

Table S4. Second order perturbation theory analysis of KS matrix in the NBO basis for unit 3 (M 2D, see Figure S5). For NBOs of lone-pair nature, the natural hybrid orbital (NHO) composition is also reported.

donor NBO (unit)	occ.	acceptor NBO (unit)	occ.	$\Delta E_{n\rightarrow\sigma^*}$ (kcal/mol)
H-bond intermolecular interactions				
$n_{N_t}(sp^{1.84}$,1)	1.86067	$\sigma^*_{N_a-H}$ (3)	0.06714	26.39
n_{N_t} $(sp^{1.84}$,2)	1.86060	$\sigma^*_{N_a-H}$ (3)	0.06724	26.43
n_{N_t} $(sp^{1.84}, 4)$	1.86073	$\sigma^*_{N_i-H}$ (3)	0.06144	26.41
$n_{N_t}(sp^{1.80}$,3)	1.86067	$\sigma^*_{N_a-H}$ (1)	0.06336	24.73
n_{N_t} $(sp^{1.80}, 3)$	1.86319	$\sigma^*_{N_a-H}$ (2)	0.06282	26.43
n_{N_t} $(sp^{1.80}, 3)$	1.86328	$\sigma^*_{N_a-H}$ (4)	0.06272	24.45
intramolecular donor-acceptor interactions				
$n_{N_a}(p)$	1.72226	$\pi^*_{C-N_t}$	0.50152	74.17
$n_{N_t}(sp^{1.80})$	1.86328	$\sigma^*_{N_t-C}$	0.03863	11.80
$n_{N_t}(sp^{1.80})$	1.86328	$\sigma^*_{N_t-C}$	0.03863	11.57
$n_{N_a}(p)$	1.72193	$\pi^*_{C-N_t}$	0.50153	74.26
$n_{N_a}(p)$	1.72205	$\pi^*_{C-N_t}$	0.50128	74.20
$n_{N_t}(sp^{1.80})$	1.86237	$\sigma^*_{N_t-C}$	0.03766	11.58
$n_{N_t}(sp^{1.80})$	1.86237	$\sigma^*_{N_t-C}$	0.03766	11.78
$n_{N_t}(sp^{1.80})$	1.86319	$\sigma^*_{N_t-C}$	0.03863	11.81
$n_{N_t}(sp^{1.80})$	1.86319	$\sigma^*_{N_t-C}$	0.03863	11.57
π_{C-N_t}	1.75936	$\pi^*_{C-N_t}$	0.50153	49.70
π_{C-N_t}	1.75942	$\pi^*_{C-N_t}$	0.50128	49.74
π_{C-N_t}	1.75925	$\pi^*_{C-N_t}$	0.50152	49.75

Table S5. Second order perturbation theory analysis of KS matrix in the NBO basis for isolated M. For NBOs of lone-pair nature, the natural hybrid orbital (NHO) composition is also reported.

donor NBO	occ.	acceptor NBO	occ.	$\Delta E_{n\rightarrow\sigma^*}$ (kcal/mol)
$n_{N_a}(p)$	1.76253	$\pi^*_{C-N_t}$	0.47970	63.00
$n_{N_t}(sp^{1.85})$	1.90623	$\sigma^*_{N_t-C}$	0.03489	12.38
$n_{N_t}(sp^{1.85})$	1.90623	$\sigma^*_{N_t-C}$	0.03489	12.38
$n_{N_a}(p)$	1.76253	$\pi^*_{C-N_t}$	0.47970	63.00
$n_{N_a}(p)$	1.76253	$\pi^*_{C-N_t}$	0.47970	63.00
$n_{N_t}(sp^{1.85})$	1.90623	$\sigma^*_{N_t-C}$	0.03489	12.38
$n_{N_t}(sp^{1.85})$	1.90623	$\sigma^*_{N_t-C}$	0.03489	12.38
$n_{N_t}(sp^{1.85})$	1.90623	$\sigma^*_{N_t-C}$	0.03489	12.38
$n_{N_t}(sp^{1.85})$	1.90623	$\sigma^*_{N_t-C}$	0.03489	12.38
π_{C-N_t}	1.74171	$\pi^*_{C-N_t}$	0.47970	50.54
π_{C-N_t}	1.74170	$\pi^*_{C-N_t}$	0.47970	50.64
π_{C-N_t}	1.74171	$\pi^*_{C-N_t}$	0.47970	50.59

Table S6. Second order perturbation theory analysis of KS matrix in the NBO basis for isolated CA. For NBOs of lone-pair nature, the natural hybrid orbital (NHO) composition is also reported.

donor NBO	occ.	acceptor NBO	occ.	$\Delta E_{n\rightarrow\sigma^*}$ (kcal/mol)
$n_{N_i}(p)$	1.67407	π^*_{C-O}	0.32367	56.28
$n_{N_i}(p)$	1.67407	π^*_{C-O}	0.32367	56.28
$n_{N_i}(p)$	1.67407	π^*_{C-O}	0.32367	56.28
$n_{N_i}(p)$	1.67407	π^*_{C-O}	0.32367	56.28
$n_{N_i}(p)$	1.67407	π^*_{C-O}	0.32367	56.28
$n_{N_i}(p)$	1.67407	π^*_{C-O}	0.32367	56.28
$n_O(p)$	1.83742	$\sigma^*_{N_i-C}$	0.08913	28.48
$n_O(p)$	1.83742	$\sigma^*_{N_i-C}$	0.08913	28.48
$n_O(p)$	1.83742	$\sigma^*_{N_i-C}$	0.08913	28.48
$n_O(p)$	1.83742	$\sigma^*_{N_i-C}$	0.08913	28.48
$n_O(p)$	1.83742	$\sigma^*_{N_i-C}$	0.08913	28.48
$n_O(p)$	1.83742	$\sigma^*_{N_i-C}$	0.08913	28.48
$n_O\ (sp^{0.59})$	1.98144	ryd.(C vicinal)	0.01039	14.00
$n_O\ (sp^{0.59})$	1.98144	ryd.(C vicinal)	0.01039	14.00
$n_O\ (sp^{0.59})$	1.98144	ryd.(C vicinal)	0.01039	14.00

S2. Evolution of N_i-H and N_a-H bond distances in going from the isolated monomers to the homo- and hetero-molecular structures.

The partial electron transfer from the lone pair of the donor heteroatom to the antibonding $\sigma^*_{N_a-H}/\sigma^*_{N_i-H}$ orbitals determine a lengthening (weakening) of the N_i-H and N_a-H bonds, the effect being proportional to the electronic charge transferred as a result of the interaction. This effect is usually counterbalanced by a rehybridization-promoted A-H bond strengthening, which in our case can be quantified by the increase in *s*-character of nitrogen natural hybrid orbital (NHO) in the N(i,a)-H bonds [2]. Both N(i,a)-H bond lengths and sp^λ valence hybrid orbitals on atom N(i,a) are reported on Table S7. In going from the free molecules to the homomolecular aggregates there is a more pronounced N-H bond elongation for CA compared to M (Table 4a). By inspecting the nature of the NHOs we see that the s-character of the Ni NHOs is lower than that corresponding to Na (Table 4b), which means that the effect of rehybridization-induced strengthening is somewhat less pronounced for the Ni-H bond. As a result of the heteromolecular H-bond network formation both N(i,a)-H bond lengths increase, albeit only moderately for the Na-H bond (in spite of a pronounced decrease of s-character of the corresponding Na NHO) while the Ni-H bond experiences a sizeable elongation (of about 0.05 Å) and a *s*-character of the corresponding Ni NHO similar to that characteristic of the homomolecular 2D structure. This observation is also in line with a larger electron transfer from the lone pair of the donor heteroatom (Nt) to the antibonding Ni-H orbital.

Table S7 N(i,a)-H bond lengths (in Å) and sp^λ valence hybrid orbitals (in parenthesis) on atom N(i,a) for free CA/M and homo- and heteromolecular aggregates.

	CA/M free	CA/M 2D	CA*M
Na-H	1.004 ($sp^{2.20}$)	1.010 ($sp^{1.93}$)	1.025 ($sp^{2.14}$)
Ni-H	1.011 ($sp^{2.31}$)	1.037 ($sp^{2.09}$)	1.089 ($sp^{2.06}$)

References

[1] G. Fronzoni, A. Cossaro, R. Costantini, L. Di Nardi, E. Viola, G. Balducci, V. Lanzilotto, D. Toffoli, Appl. Surf. Sci. 2024, 642, 158573.

[2] I. V. Alabugin, M. Manoharam, S. Peabody, and F. Weinhold, J. Am. Chem. Soc. 2003, 125, 5973-5987.
